# Treatment of Gaseous Gangrene by the Use of Serum

**Published:** 1917-12

**Authors:** 


**Treatment of Gaseous Gangrene by the Use of Serum.** Abstract of a Portion of a Lecture on "Bacteriological and Experimental Researches on Gas Gangrene" by M. WEINBERG, published in the *Proceedings of the Royal Society of Medicine*, 1916, Vol. IX, p. 119-144.

The author states that at the beginning of the war he and his associates prepared an anti-Bacillus perfringens (aerogenes capsulatus) vaccine which seemed to yield good results in cases of subacute gas gangrene in which the B. perfringens was the most pathogenic organism. The author believes, however, that the best vaccine would be one prepared with all the organisms, both aerobes and anaerobes, to be found in the wounds. Such vaccine could not be prepared by the classic method, because the spores resist the temperature, but it has been produced by treating the mixture of organisms with iodine. Since the vaccine should be used at the earliest possible moment, the author prepares the omnivalent iodized autovaccine from the wound discharge with a delay of not more than two hours. Several injections are made daily or every two days. Later, if thought best, an autovaccine may later be prepared with cultures of the isolated organisms.

W. believes that this treatment should be employed as a supplement to the large excision and antiseptic methods, but he believes that it has yielded good results.

The author states that to meet the more fatal types of the infection, he is attempting to prepare an active serum with the three most pathogenic organisms of gangrene : Bacillus perfringens, the Vibrion septique, and Bacillus œdematiens.

W. tells of a serum obtained from horses after Bacillus perfringens had been injected in living doses for a year. Although the activity of this serum is comparatively small, it has given excellent results in a number of cases, although it appears powerless against septicemia.

Similarly antitoxic sera of the Vibrion septique and B. œdematiens have been prepared. On the whole the results of such experiments have been disappointing, and the sera difficult to obtain. Nevertheless the author expects that solutions will be found to all the difficulties, and that sera will be obtained which, like the antitetanic serum, may not be reliable as a curative measure, but which, used as a preventive measure, will give valuable results. He believes that a mixture of the sera for the three chief organisms mentioned above, injected in the wound and in the neighboring tissues will be the ideal preventive treatment. Towards this end he calls upon all bacteriologists to cooperate.

(Note that in the later article, p. 95, the author speaks of a mixed serum which he has obtained, and which he recommends by way of a routine preventive treatment for all wounds.)

THE USE OF THE SERUM OF LECLAINCHE AND VALLÉE.

Weinberg speaks favorably also of the use of Leclainche's and Vallée's polyvalent serum, especially in wounds infected with streptococci. The use of this serum for gas gangrene is mentioned favorably by M. Quenu, M. L. Bazy, and M. Routier in a discussion of a paper on the " Reappearance of gaseous gangrene in Secondary

Amputations " (*Archives de Médecine et de Pharmacie Militaires*, March, 1917, p. 402).

For further results obtained by sera, see the conclusions on *Gaseous Gangrene* drawn up by the 3rd Inter-allied Surgical Conference, p. 75. ; M. Sacquépée read a paper at the Conference upon which these conclusions are based.

